# Evaluating the lexico-grammatical differences in the writing of native and non-native speakers of English in peer-reviewed medical journals in the field of pediatric oncology: Creation of the genuine index scoring system

**DOI:** 10.1371/journal.pone.0172338

**Published:** 2017-02-17

**Authors:** Alberto Alexander Gayle, Motomu Shimaoka

**Affiliations:** 1 Center for Medical and Nursing Education, Mie University School of Medicine, Mie, Japan; 2 Department of Immunology, Mie University Graduate School of Medicine, Mie, Japan; 3 Department of Molecular Pathobiology and Cell Adhesion Biology, Mie University Graduate School of Medicine, Mie, Japan; 4 Center for Disaster Medicine Research and Education, Mie University Graduate School of Medicine, Mie, Japan; Garvan Institute of Medical Research, AUSTRALIA

## Abstract

**Introduction:**

The predominance of English in scientific research has created hurdles for “non-native speakers” of English. Here we present a novel application of native language identification (NLI) for the assessment of medical-scientific writing. For this purpose, we created a novel classification system whereby scoring would be based solely on text features found to be distinctive among native English speakers (NS) within a given context. We dubbed this the “Genuine Index” (GI).

**Methodology:**

This methodology was validated using a small set of journals in the field of pediatric oncology. Our dataset consisted of 5,907 abstracts, representing work from 77 countries. A support vector machine (SVM) was used to generate our model and for scoring.

**Results:**

Accuracy, precision, and recall of the classification model were 93.3%, 93.7%, and 99.4%, respectively. Class specific F-scores were 96.5% for NS and 39.8% for our benchmark class, Japan. Overall kappa was calculated to be 37.2%. We found significant differences between countries with respect to the GI score. Significant correlation was found between GI scores and two validated objective measures of writing proficiency and readability. Two sets of key terms and phrases differentiating NS and non-native writing were identified.

**Conclusions:**

Our GI model was able to detect, with a high degree of reliability, subtle differences between the terms and phrasing used by native and non-native speakers in peer reviewed journals, in the field of pediatric oncology. In addition, L1 language transfer was found to be very likely to survive revision, especially in non-Western countries such as Japan. These findings show that even when the language used is technically correct, there may still be some phrasing or usage that impact quality.

## Introduction

The biomedical sciences are presently experiencing a “golden age” [1]. Recent studies have shown that not only are research teams larger than in previous eras [2], but they are increasingly likely to be more globalized in their constitution as well [3]. At the same time, in many areas of practice and research, multi-lateral collaboration is already common place and is quickly becoming the norm [4–6]. Although English has quietly become accepted as the *lingua franca* for biomedical publication and collaboration [7–10], this development has not been without its detractors. Some argue that the predominance of English in scientific publication has created hurdles for “non-native speakers” (nNS), for whom English is a second language [10–12]. Such hurdles represent substantial additional burdens for nNS researchers—burdens that many “natives speakers” (NS) may fail to consider; these include: 1) expenses related to publisher-required professional editing [13]; 2) longer, more cumbersome drafting and revision processes [14]; and 3) difficulty describing their work with appropriate precision or nuance [15]. In addition, nNS researchers are more likely to have their research rejected for publication and feel disadvantaged by or less satisfied with the peer-review process [16]. Consequently, to ensure that research achievements of nNS are appropriately applied and recognized, more time-efficient and practical methods to improve the drafting and publication capabilities of nNS researchers must be developed.

Native language identification (NLI) refers to the lexico-grammatical and stylistic analysis of a text to determine the native language of the author [17]. It is considered to be a sub-genre of natural language processing [18]. As Koppel et al [17] described, this is accomplished by identifying idiosyncratic patterns in word choice, phrasing, and errors. Standard text mining procedures are applied to obtain and process collections of appropriate text data (“corpora”), which are ultimately converted into vector representations [19] allowing a full range of descriptive and predictive statistical analyses, including association, clustering, and classification [20]. In the case of NLI, the most common analytical method applied is classification analysis, whereby pre-labeled groups of documents are analyzed in order to develop a statistical model capable of reliably predicting category assignment [18,21]. In traditional multivariate classification (e.g., logistic regression), the category to be assigned would be described as the independent variable. For the purpose of NLI, given a corpus containing text written in a common L2/second language (e.g., English), the classification category to predict will be the L1/native language of the writer. Dependent variables used to predict the L1 have typically been lexico-grammatical features such as part-of-speech usage, n-gram sets of characters or words, functional word usage, vocabulary content, and errors in spelling and grammar [22]. And just as in the case of logistic regression, these variables can then be selected or excluded based on performance and explanatory power [23].

Over the past decade since its inception, NLI has been applied in many contexts. These range from forensic analysis of anonymous text, to educational applications such as automated writing assessment [24]. In the latter case, L1-specific usage and error patterns are identified in L2-text to inform assessment [25]. The practicality of NLI has largely been attributed to the phenomenon of “interlanguage” [26], which is the supposition that adult nNS are relatively inflexible in their thinking (“fossilization”) and thus inadvertently adopt a set of conversion rules that map patterns of language from their native language (“language transfer”) [27] on to the target language that are often inconsistent with native usage (“language interference”) [28]. Accordingly, the analysis and classification of such interlanguage has also been used to develop teaching material/methods specific for a given mother tongue [29]. In the present study, we present a novel application of NLI for the assessment of medical-scientific writing. For this purpose, we have created a novel classification system based on the antithesis of the “language transfer” criteria: classification would be based solely on text features found to be distinctive among NS of English within a given context. In addition, a further aim was to develop a robust method for scoring such biomedical texts according to the level of fluency, as determined by the lexico-grammatical similarity to text written by NS within the same field. We dubbed this the “Genuine Index” (GI).

Manuscripts written by nNS authors are usually extensively corrected by NS editorial services prior to being submitted for publication in biomedical journals. Indeed, many journals require that manuscripts be corrected by NS editorial services. And although the editing and review process may eliminate or obfuscate the L1 characteristics of an nNS researcher, the impact of such quality-control processes has yet to be quantified [30]. The GI method presented here was devised primarily to address this question. We set out to validate this methodology using a small set of medical specialist journals. In the process, we were able to conclusively demonstrate that L1 language transfer is very likely to survive revision and peer-review, especially in non-Western countries such as our native Japan.

## Materials and methods

The following is summarized in S1 Fig.

### Data collection

In this study, “native speakers” are defined within the context of Kachru’s “World Englishes” [31] framework, which considers the historical and cultural context in which Enlgish is spoken. This framework defines NS countries as those in which English is and has generally been the first language. The NS countries are considered to be norm-providing with respect to the remainder [32]. This definition includes the United States, United Kingdom, Canada and Australia. Accordingly, we developed a classification model capable of identifying and characterizing idiosyncrasies unique to authors hailing from these NS countries.

For this proof-of-the-principle study, we limited our search to the two leading journals representative of a field in which the authors had sufficient expertise, pediatric oncology: 1) Pediatric Blood & Cancer (ISSN#: 1545–5017, impact factor: 2.386), and 2) Pediatric Hematology and Oncology (ISSN#: 1521–0669, impact factor: 1.096). Data was obtained from PubMed using the following search criteria: “Pediatr Blood Cancer”[Journal] OR “Pediatr Hematol Oncol”[Journal] AND “has abstract”[text]. This search yielded 5,907 abstracts, representing work published by authors in 77 countries between 1986–2015. For control purposes, this dataset was supplemented to include another two leading journals representative of a reasonably distinct field in which the authors also had experience, anesthesiology: 1) Anesthesia and Analgesia (ISSN#: 1526–7598, impact factor: 3.827), and 2) Anaesthesia (ISSN#: 13652044, impact factor: 3.794). Search criteria for this supplementary sample was aligned with the primary sample in terms of coverage dates and representative geographies to reduce undue bias: "Anaesthesia"[Journal] OR "Anesthesia and analgesia"[Journal] AND (hasabstract[text] AND ("1986/01/01"[PDAT]: "2015/12/31"[PDAT])). This search yielded an additional 16,952 abstracts. This data has been uploaded for reference under the following cited Github repository [33].

Abstract data was downloaded from PubMed and converted into data tables using a custom program and processing algorithm developed using RapidMiner Studio 6.5 (RapidMiner GmbH. Released 2015. RapidMiner Studio Academia, Version 6.5002). RapidMiner facilitates the creation of custom algorithms and workflows, in addition to providing a common set of analytical tools for text and data analytics. The resulting data table included the abstract text and the stated affiliation of the first author.

The International English Language Testing System (IELTS) is one of the most widely recognized standards for evaluating English language competence [25] and is regularly included as a requirement for non-native English speaking students and medical professionals wishing to study and/or work in countries where English is the first language [34,35]. This examination includes separate listening, reading, writing, and speaking components [36]. The IELTS website publishes aggregate test scores annually. We downloaded and extracted the most currently available version of this data (2014) for each country, where available.

### Data processing

A custom algorithm was applied to identify and extract country information based on the affiliation of the first author. This is an automated version of a methodology that has been used successfully in the previous works that studied the relationship between English proficiency and publication output [11,37]. Application was further justified by prior research demonstrating that the first author is likely to be the primary and definitive contributor in any given publication [38]. Country information was then manually checked to ensure consistency of naming conventions and for accuracy. Twenty-eight abstracts lacked first author affiliation data, and thus no country information was extracted. The extracted country information was appended to the data table. We assigned four countries, the United States, the United Kingdom, Canada, and Australia, to be representative of the “native speakers”, and labeled each abstract accordingly.

Abstracts were processed in order to derive term statistics suitable for quantitative analysis. The initial processing steps involve parsing strings to identify individual *word tokens* which are then programmatically homogenized so as to ensure equivalent word forms are not double counted [40]. The Porter stemming algorithm [39] was use for this purpose, together with a custom script to standardize spelling to US American norms. For this analysis, contiguous token sets (*n-grams*) ranging in length from one word to four word phrases were generated and included in the data set. Despite evidence to suggest that character-based n-gram models may perform marginally better in native language identification applications [41,42], word n-grams were selected in order deliver a more parsimonious model that would allow for a more mechanistic interpretation of results. For our purposes, all n-grams appearing in more than 20% of all abstracts as well as those appearing in less than 2% were pruned. This was done for the dual purpose of reducing the set of data to be analyzed and filtering out tokens, both common and rare, unlikely to contribute to our primary task of lexico-grammatical differentiation. These thresholds were chosen following an inconclusive literature review to determine optimal pruning thresholds [43]. TFIDF, which is an acronym for “term frequency inverse document frequency”, is an n-gram weighting criteria that has been found to improve the performance of NLI tasks [44]. It is defined as follows: $\text{TFIDF }=\;tf*\text{log}\left(\frac{N}{df}\right)$, where *tf* is the frequency of a term within a given document, *df* is the frequency across all documents, and *N* is the total number of documents. This weighting scheme emphasizes the importance of key but not uncommon terms [45–47]. The result of this process is a corpus composes of *word vectors* indicative of the TFIDF of each 2–20% incident n-gram, for each respective abstract.

### Data analysis

Following the vectorization process described above, a classification model was trained to generate our GI scoring model. For our purposes, we opted to use a classifier based on the support vector machine (SVM) algorithm. SVM is considered to be one of the most powerful analytical options in the field of biomedical informatics [48] and is increasingly being preferred over more traditional, regression-based methods [49,50], particularly for classification tasks in which a minimal understanding of the underlying structure is required [51–53].

Support vector machines are supervised discriminative classifiers that attempt to define a hyperplane that maximizes the separation between the input cases (i.e., word vectors) with respect to a predefined binary class (e.g., native speaker status) [54]. To achieve maximum separation, cases are mapped to increasingly higher-dimensional spaces until a linear solution is found. The result is then transformed back down to the original dimensions. As a result of this process, the SVM-generated model is not readily interpretable, as the boundaries defined by the algorithm are likely to be non-linear when projected back into the original space [49,55]. However, SVMs do have the unique advantage of assigning weights to each of the input features (i.e., n-grams) that correspond with their respective coordinates along the hyperplane. This allows the direct assessment and comparison of the relative importance of each with respect to the generated model [56]. However, inferring too much from the weights alone, without regard for the underlying linear associations, is generally ill-advised [57]. Accordingly, for our purposes, weights were considered only with respect to their ordinal values.

Default settings were used for the support vector machine. For model training and validation, a stratified sampling and cross-validation process was employed. In this process, randomized but statistically homogeneous subsamples are selected and used to train the model. Each subsample is balanced to ensure that the class distribution matches the entire sample [58]. The remaining sample is then used to test and validate the previously-derived model. This process is conducted in an iterative fashion, according to user-defined criteria, and aggregated results are returned [51]. This entire process is automated within RapidMiner.

In our case, cross-validation was conducted 10 times with randomized training sets equal to 10% of the native-speaker/Japanese sample. The resulting model, along with all statistically relevant n-grams, were then returned and saved. This model was then applied to the remaining abstracts in our dataset to predict classification assignments based on the previously derived model. This process generated a “confidence” metric indicative of the probability (0–100%) that a given abstract was written by a native speaker. This probability was based on the respective distance of each abstract from the modeled hyperplane [59,60]. This confidence metric was then multiplied by 100 and then rounded to yield our “Genuine Index” (GI) score. For example, an abstract determined by the GI algorithm to hold a 69.2% likelihood of being NS would receive a GI score of 69. The GI scoring process was then run against the original dataset, to assess and score the relative “genuineness” of each of the contributing abstracts with respect to the NS ideal implied by our model.

Once a GI score was generated for each abstract, the dataset was aggregated according to country, and those with more than n = 40 abstracts were retained for further analysis (S1 Fig). This cut-off value was chosen to ensure the assumption of normality [61] for subsequent statistical tests. Visual inspection of histograms for each included country confirmed the validity of this convention (S2 Fig). For each country, the aggregate GI score was assessed for statistically significant variation within and across groups using the 1-way ANOVA and post-hoc tests. Analyses were conducted at the 95% level using SPSS 23 (IBM SPSS Statistics for Windows, Version 23.0).

For validation purposes, we evaluated the relationship between GI score and readability, as determined by the Coleman-Liau Index (CLI). The CLI is a bibliometric measure based on the analysis of word and sentence-level complexity. CLI is an established, validated metric and has been demonstrated to reliably predict the grade-level readability of a given English text [62] and has been used recently in similar studies [63]. This formulation was derived based on research demonstrating that average letter count per word and average word count per sentence are reliable predictors of grade-level readability. For a given text, CLI is derived as follows: $CLI=5.89\left(\frac{\text{c}}{\text{w}}\right)-29.5\left(\frac{\text{s}}{\text{w}}\right)-15.8$, where *c* is the character count, *w* is the word count, and *s* is the sentence count. Further validation was provided by testing for association between the aggregate GI scores and aggregate IELTS scores, where available. All analyses were conducted using SPSS 23.

### Evaluation & performance criteria

Classification performance was evaluated using a type of contingency table referred to as a confusion matrix. Given m classes, the confusion matrix is an m x m table that compares the predicted classification results with the actual input class, as defined *a priori*. Based on the confusion matrix, classification results can thus be characterized as true positive (TP), false positive (FP), true negative (TN), and false negative (FN). These values can then be used to calculate the following performance metrics: 1) precision (positive predictive value), which reflects the likelihood that a given class prediction will be correct: $p=\;\frac{TP}{TP+FP}$; 2) recall (sensitivity), which reflects the likelihood that abstracts belonging to a given class will be accurately classified: $r=\frac{TP}{TP+FN}$; and 3) F-measure, which is a holistic measure that takes into account both precision and recall: $f=\frac{2\text{pr}}{\text{p}+\text{r}}$. RapidMiner automatically calculates precision and recall; Excel was used to manually calculate the F-measure.

## Results

### Genuine index score

This study tested the Genuine Index Scoring System on a relatively limited subject-area: pediatric oncology. Our selection of this subject-area was influenced by the practical expertise of the authors. In the field of pediatric oncology, treatment is standardized at the national level, following an annual review of national and international data. Accordingly, the themes discussed and the technical terminology used are expected to be reasonably homogenous on a country-to-country basis. In addition, the limited range of diagnostic and patient-related characteristics in this field were expected to ensure a further level of homogeneity as well.

We included 77 countries in this dataset (Table 1). On the aggregate level, our Genuine Index model performed exceptionally well in terms of overall accuracy, precision, and recall (93.3%, 93.7%, and 99.4% respectively). However, when the Japanese and nNS classes were examined more closely, substantial variation was found. The GI model correctly identified Japanese authors in 81% of the cases classified as Japanese (n = 79); while in those cases identified by our model as NS (n = 2829), the GI model correctly identified NS authors (i.e., those from the US, UK, CA, and AU) 94% of the time (Table 2). However, overall, Japanese authors were correctly identified by the GI model in only 26% of cases (n = 243), possibly because the editing and review process might partially obfuscate the L1 characteristics of nNS Japansese authors. This contrasts sharply with NS, who were able to be correctly identified by our model in 99% of cases overall (n = 2665) (Table 2). For NS, our model produced an F-score of 96%; whereas for abstracts affiliated with Japan, the F-score was only 40% (Table 2, footnote). This yielded an overall model accuracy of 93.3% and a kappa of 37.2%.

**Table 1:** Number of abstracts per country.

Country	Abstracts
USA	2085
Turkey	476
Italy	283
Germany	273
Canada	272
UK	262
Japan	244
The Netherlands	161
France	145
Israel	139
India	136
China	103
Sweden	87
Greece	86
Brazil	78
Australia	71
Austria	63
Denmark	55
Iran	54
Switzerland	54
Finland	54
Poland	53
South Korea	48
Spain	46
Taiwan	45
Egypt	42
Norway	42
Belgium	34
South Africa	33
Former Yugoslavia	33
Hungary	26
Argentina	26
Hong Kong	24
Czech Republic—Slovakia	22
Saudi Arabia	21
Mexico	17
Oman	16
Nigeria	15
Jordan	15
Lebanon	13
Portugal	10
Thailand	10
Other	87
Unidentified	28
**Total**	**5907**

Number of abstracts per country, obtained from Pediatric Blood & Cancer and Pediatric Hematology/Oncology, 1986–2015. Countries with with less than 10 abstracts aggregated under “Other”. Abstracts with unidentified countries aggregated under “Unidentified”.

**Table 2:** Genuine index model performance.

	True JPN (n = 243)	True NS (n = 2665)	Precision
**Predicted JPN (n = 79)**	64	15	81.0%
**Predicted NS (n = 2829)**	179	2,650	93.7%
**Recall**	26.3%	99.4%	

Confusion matrix detailing classification results of NS and Japanese researchers, where each cell represents number of corresponding abstracts. Includes performance metrics derived from confusion matrix, in which larger numbers represent better performance: F-score denotes performance for each respective class, while kappa denotes performance for the overall model. Class specific F-score calculated based on the above: JPN = 39.8%, NS = 96.5%. Overall kappa calculated based on the above: 37.2%.

**Fig 1:**
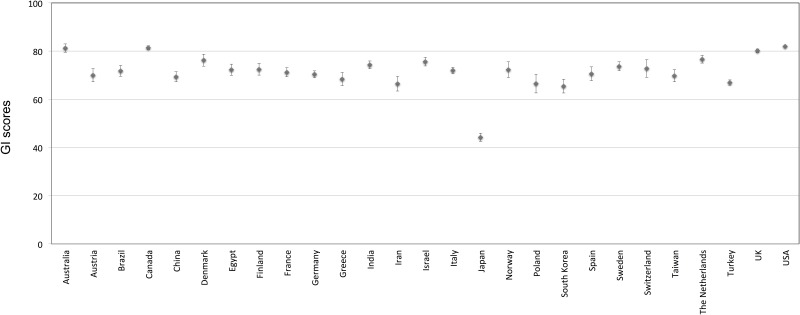
95% confidence intervals for GI score by country (n > 39 abstracts). X-axis denotes countries according to which GI score is aggregated. Y-axis denotes mean GI score per country. Means and 95% confidence intervals for each country reveal substantial variation, albeit with most averages falling within the 60–80 range.

We found significant differences between NS, nNS and the countries retained for further analysis with respect to the GI score. For the NS group, mean GI score was quite high at 81.7, with a total range of 47 to 96. For the Japan-affiliated authors, however, mean GI score was almost half that of the native speakers (44.3) (Fig 1, Table 3), with a total range of 13 to 76 (S2 Fig). For the other, remaining nNS countries, the GI score ranged from 23 to 94, with a mean of 71.3 (Fig 1, S2 Fig). Overall, the GI score was found to range from 96 to 13.

**Table 3:** Identification of homogenous subsets among countries with respect to GI score.

Country	N	1	2	6	7	8	9	10	11	12	13
Japan	244	44.3									
South Korea	48		65.5								
Iran	54		66.5								
Poland	53		66.5								
Turkey	476		67.0								
Greece	86		68.4	68.4							
China	103		69.4	69.4	69.4						
Taiwan	45		69.8	69.8	69.8						
Austria	63		70.0	70.0	70.0						
Germany	273		70.4	70.4	70.4	70.4					
Spain	46		70.6	70.6	70.6	70.6					
France	145			71.2	71.2	71.2	71.2				
Brazil	78			71.8	71.8	71.8	71.8	71.8			
Italy	283			72.0	72.0	72.0	72.0	72.0			
Egypt	42			72.3	72.3	72.3	72.3	72.3			
Norway	42			72.3	72.3	72.3	72.3	72.3			
Finland	54			72.5	72.5	72.5	72.5	72.5			
Switzerland	54			72.8	72.8	72.8	72.8	72.8			
Sweden	87			73.7	73.7	73.7	73.7	73.7			
India	136				74.3	74.3	74.3	74.3			
Israel	139					75.6	75.6	75.6	75.6		
Denmark	55						76.3	76.3	76.3	76.3	
Netherlands	161							76.6	76.6	76.6	
UK	262								80.1	80.1	80.1
Australia	71									81.3	81.3
Canada	272									81.4	81.4
USA	2085										82.0
Sig.		1.00	0.07	0.05	0.11	0.06	0.08	0.13	0.24	0.07	1.00

Table of differences between means generated by Tukey HSD post-hoc test with statistical differences highlighted. Means for groups in homogeneous subsets are displayed. a. Uses Harmonic Mean Sample Size = 81.905. b. The group sizes are unequal. The harmonic mean of the group sizes is used. Type I error levels are not guaranteed.

Intercountry differences were considered next. Visual inspection of a histogram for each country’s respective GI score distribution confirmed each to be approximately normal (S2 Fig) and Levene’s test confirmed equality of variances (p = 0.00). An ANOVA confirmed there to be significant differences between the selected countries with respect to GI score (p = 0.00). The *post-hoc* Tukey-Kramer test for homogeneous subsets revealed that native speaking countries were statistically equivalent with respect to GI score (p = 1.00) and, as a group, scored significantly higher than all other countries (US 82.0, Australia 81.4, Canada 81.3, and the UK 80.1), with The Netherlands, Denmark, and Israel forming a close second tier with mean score distributions that were statistically indistinguishable from that of the UK (76.6, 76.3, and 75.6 respectively; p = .24). The remaining countries, with the notable exception of Japan, formed a third tier with GI scores that too were generally insignificant with respect to each other (Table 3). Mean GI scores for the US and Canada were found to be significantly higher than all other second and third tier countries, while the UK and Australia performed better than most other second and third tier countries save the Netherlands and Denmark. The GI score for Japan was found to be significantly lower than all other countries. All significance tests were performed at the p = .05 level. S1 Table includes the results of the means tests for all country combinations.

**Fig 2:**
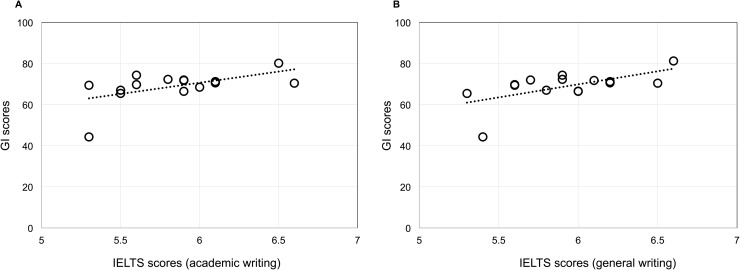
Analysis of correlation between national IELTS results and GI scores (n = 15). Pearson correlation demonstrates statistically significant, albeit minor, correlation between between aggregate GI scores and IELTS scores for the countries where data exists. (A), academic writing: correlation = 0.5485, p = 0.034. (B) general writing: correlation = 0.6009, p = 0.018).

The GI score was found to be positively correlated with reading complexity, as measured by CLI (correlation coefficient: 0.031; p = 0.021); a regression analysis determined the strength of association to be 0.144 (Table 4). Overall, CLI ranged from 5.7 to 29.6 with a mean of 16.9. In addition, we found minor yet significant correlation between the GI score and IELTS writing scores. As shown in Fig 2, the result of a Pearson correlation was found to be significant for both variations of the IELTS, Academic (correlation: 0.55; p = 0.34) and General Training (correlation: 0.60; p = 0.18).

**Table 4:** Regression analysis: Coleman-Liau index vs GI score.

		Unstandardized Coefficients			
Model		B	Std. Error	t	Sig.
1	(Constant)	72.622	1.070	67.857	0.000
	CLI	.144	.063	2.303	.021

Table shows the output for a regression analysis in which CLI is modeled as the dependent variable and GI score as the independent variable. Results suggest an association between increasing text complexity (CLI) and a writing style closer to that of the ideal (higher GI score). These results could be interpreted to imply that a 6.9 point increase in GI score is equivalent to a 1 year increase in grade-level; however, further research would be needed to substantiate.

### Terms characteristic of native speakers in pediatric oncology

As previously discussed, our model also calculated weights corresponding to each n-gram’s position along the hyperplane, with larger values indicating greater discriminatory distance. Table 5 displays the n-grams with the highest weights for differentiating the writing of NS and nNS researchers, in our model. The implications of these findings are discussed below.

**Table 5:** Top terms according to discriminatory power (SVM weights).

Terms for Japanese (nNS)	Weight	Terms for NS	Weight
showed	0.060	child	0.044
although	0.056	transplant	0.036
detected	0.056	found to	0.033
serum	0.055	patients had	0.029
after	0.050	secondary	0.028
having	0.050	anemia	0.028
analyzed	0.048	agents	0.027
because	0.046	protocols	0.026
without	0.045	hematologic	0.025
old	0.038	post	0.025
that the	0.037	negative	0.024
the patient	0.036	review	0.023
transplantation	0.035	demonstrated	0.023
however	0.035	consistent	0.023
year old	0.034	compared to	0.023
was performed	0.034	evaluated	0.023
remission	0.034	reviewed	0.023
infection	0.034	well	0.022
cell transplantation	0.033	diagnosed with	0.022
stem cell transplantation	0.032	receiving	0.022
mutation	0.032	other	0.022
cases	0.031	we present	0.022
course	0.031	literature	0.021
with acute	0.030	commonly	0.021
should be	0.029	effects of	0.021
age of	0.029	all patients	0.021
after the	0.029	previously	0.021
diagnosed	0.028	risk of	0.021
acute	0.027	common	0.021
followed by	0.027	survival	0.021

Ranked list of n-grams showing which terms are most significant for differentiating the writing of NS and Japanese researchers in this study. Due to the characteristics of SVM models, weights should only be interpreted as ordinal rankings; no linear relationship should be inferred.

## Discussion

In this study, the terms and phrasing used by non-native speakers of English (nNS) publishing in the area of pediatric oncology were found to differ significantly from that of their English native speaking (NS) counterparts, despite having presumably undergone professional editing and rigorous peer review. Our findings strongly support the notion championed by Pérez-Llantada [28] that cultural idiosyncrasies invariably influence the lexis and style employed by nNS writers, even when their writing is technically correct. The question as to whether and to what extent this impacts subjective “quality” was not addressed. However, it has been widely suggested that ill-formed or unfamiliar phrasing negatively impacts objective measures such as intelligibility and information retention [64–66]. And in this study, we did indeed find significant association between GI score and readability, as determined by the Coleman-Liau Index (CLI) metric.

### Examination of representative manuscripts

To underscore the significance of our findings, we present here a brief sampling of 8 abstracts, with two examples from each of the following categories: top-ranked NS countries, top-ranked nNS countries, bottom-ranked NS countries, and bottom-ranked nNS countries.

#### Example top-ranked manuscripts from NS countries

*A prospective cohort of children with sickle cell disease (SCD) was evaluated to determine the variability of daytime pulse oximetry among three measurements over approximately 1 year. Fifty-eight participants were evaluated. Asymptomatic children with initial oxygen saturation < or = 92% had a mean range over 1 year of 4.6% (2.1–7.5%). In contrast, asymptomatic children whose oxygen saturation was >92% had a mean range of 1.9% (0–5.5%). These results suggest, changes in pulse oximetry measurement of approximately 5% may not be clinically significant in otherwise, healthy children with SCD with previous pulse oximetry < or = 92%*.

(USA; 99 GI)

*Tuberous sclerosis complex (TSC) is associated with hamartomatous growths including subependymal giant cell astrocytomas (SEGAs). Since chemo-radiation therapies offer scant benefit, oncologists had traditionally been little involved in managing SEGAs. Recent evidence demonstrating rapamycin efficacy in adults and children with TSC-associated tumors foresee a practice change. We summarize our institutional experience and literature review that highlight potential benefits and hazards of rapamycin therapy, for TSC patients with SEGA, and other syndromal brain tumors*.

(Canada; 97 GI)

#### Example top-ranked manuscripts from nNS countries

*Neutropenia is a less commonly encountered feature of Rh hemolytic disease of the newborn, and its management may be problematic. Two newborn infants with neutropenia complicating Rh incompatibility-induced hydrops fetalis were treated with intravenous recombinant human granulocyte colony-stimulating factor (rhG-CSF), 5 micrograms/kg/day for 5 days. Both patients responded to therapy with a rapid and persistent increase of their neutrophil counts to normal values. The treatment was well tolerated and no adverse clinical events were observed. rhG-CSF induces a significant increase in peripheral absolute neutrophil counts of neutropenic neonates with Rh hydrops fetalis and was well tolerated. The contribution of rhG-CSF to clinical recovery warrants further investigation*.

(Israel; 89 GI)

*Patients with sickle cell disease (SCD) have an increased risk of invasive bacterial infection because of hyposplenism. Bordetella holmesii is a recently described Gram-negative coccobacillus with an apparent predilection for asplenic hosts. We report two patients with SCD and B. holmesii bacteremia. Fastidious growth in culture and a typically uncomplicated clinical course distinguish B. holmesii infection from other invasive bacterial infections in SCD. Providers for patients with SCD should be aware of this pathogen and ensure that their microbiology laboratories are capable of isolating and identifying this organism*.

(Netherlands; 90 GI)

#### Example bottom-ranked manuscripts from NS countries

A 3-year-old female was diagnosed with acute myeloid leukemia (AML-M2). The disease was refractory to various chemotherapeutic agents. Cytogenetic analysis revealed a clone with trisomy 8 at diagnosis that was replaced by a clone containing a t(11;15) and del(20q) by the end of the second induction. A new clone, characterized by a Philadelphia chromosome, with the minor BCR/ABL p190 transcript, emerged 14 months after diagnosis and remained to the end of disease course. The late occurrence of the Philadelphia chromosome in AML has been documented rarely in adults.

(Canada; 51 GI)

We report the case of a 6-year-old male who was referred to a tertiary oncology center with a focal brainstem lesion which was presumed to be neoplastic. Due to the symmetric nature of the lesion on magnetic resonance imaging, the evaluation was expanded to investigate other possible causes and eventual diagnosis of Alexander's disease (AD) was made. AD is a neurodegenerative disease which must be included in the differential for tumor-like lesions within the posterior fossa.

(USA; 46 GI)

#### Example bottom-ranked manuscripts from nNS countries

Combined deficiency of coagulant activity of the vitamin K-dependent factors was found in a 14-year-old boy suffering from severe hemorrhages. Immunoassays revealed the presence of acarboxyprothrombin. The bleedings could be controlled, but the coagulation defects persisted during more than 2 years' follow-up and could not be corrected by oral or parenteral vitamin K. No intoxication or underlying disease was found. The abnormality was considered a congenital disorder of the carboxylation of prothrombin.

(Sweden; 40 GI)

We examined 52 children with advanced neuroblastoma who were diagnosed and treated during the past 7 years, and investigated the correlation between the degree of lymph node (LN) metastasis and the prognosis of neuroblastoma. In 8 of the 52 patients, distant LN metastasis was confirmed both radiographically and histologically. The urinary homovanillic acid (HVA) level was markedly elevated in these patients, and it was higher than that in patients with regional LN metastasis (p less than .05). The urinary vanillylmandelic acid (VMA) level and the VMA/HVA ratio were not significantly different between patients with regional and distant LN metastasis. None of the four examined patients with distant LN metastasis showed N-myc amplification of neuroblastoma tumors. An analysis of the survival rate in each patient group classified according to the degree of LN metastasis showed that the prognosis of the patients without LN metastasis or with distant LN metastasis tended to be better than that of patients with regional LN metastasis. Our results indicate that patients with distant LN metastasis may belong to a subclass with different biological features and a better prognosis than those of other groups.

(Japan; 38 GI)

### GI model implications

As we began this study, we were concerned that the copy-editing process might potentially limit the range of linguistic variability detectable by NLI-based methods. This is a reasonable concern given that the process of revision and copy-editing by the authors and/or the Journals is designed to effectively standardize all publication output with respect to linguistic style and quality. Despite the potential limitation, our “Genuine Index” (GI) model was found to have generated a good dynamic range, with strong, statistically significant differences between countries. Furthermore, the score generated by our GI model was found to correlate significantly with standardized English writing scores and established measures of readability. This adds further validation to the power of our GI method by confirming that the underlying dimensions being assessed are indeed related to English language proficiency and output quality.

SVM-based models such as ours are limited in the sense that linear interpretation is not possible. Indeed, we were able to identify terms and phrases that differentiate the edited, peer-reviewed publications of nNS and NS researches in the field of pediatric oncology (Table 5). For example, where NS used the term “child” to describe pediatric patients, Japanese authors were most likely to use “the patient”. Other examples include: 1) “transplant” (NS) versus “transplantation” (Japanese authors), and 2) “found to” and “demonstrated” (NS) versus “showed” (Japanese authors). However, the specific relationship between the various terms and phrases included in our model remains undefined. Are the problems of nNS researchers related more to lack of lexical variety, or is more related to the usage of indigenous English constructions, or is it more a case of substandard grammar in general?

While an SVM-based analysis provides little information in this respect, a qualitative examination of key term differences demonstrates that the key constructions being used by nNS are not unintelligible, but instead likely due to a reduced awareness of socio-professional norms.

### Corpus considerations

In corpus-derived analyses such the present one, the quality and comprehensiveness of a given corpus directly impacts the quality and reliability of the results. This is analogous to the concept of sample representativity in traditional, multivariate analyses where data quality is emphasized over quantity (i.e., “big data”). This study demonstrates that, just as in the case of multivariate analyses, analytical robustness can be improved by carefully controlling corpus characteristics. This is analogous to the concepts of quota sampling or sample weighting in multivariate analyses, which are well-established methods for improving analytical robustness for a given sample size. Accordingly, we decided *a priori* to control corpus characteristics as follows: 1) include only abstracts rather than full texts; 2) exclude data presumed to be of analytically lesser relevance via pruning; and 3) restrict the topic to a single, limited subdomain.

This study was designed to construct a model and scoring system for identifying idiosyncrasies in the technical language and phrasing common in peer-reviewed biomedical publications. Basic grammar and collocational usage were specifically omitted from consideration. This is reflected in our decision to include only abstracts, which are known to be the most highly polished and technically dense section in any given publication [67]. The language present in abstracts was thus assumed to be the most representative of language key to a given biomedical domain. To further ensure domain representativity, we omitted individual terms and n-grams appearing in more than 20% of all abstracts and those appearing in less than 2%. This step, while common across various types of text-based classification tasks [68], is notably absent from the NLI literature. This is not surprising given that higher incidence terms and phrasing (e.g., articles and prepositional phrases) have been demonstrated to be the sources of most grammatical errors typical of nNS [21,25]. The lower, 2% cut-off was implemented so as to minimize any bias due to region-specific topic trends. These thresholds were implemented following an inconclusive literature review and are subject to revision; further research will be required to determine the optimal pruning thresholds with respect a) the medical-scientific literature domain, b) model performance, and c) model interpretability. In addition, to further reduce potential for systemic variation unrelated to language considerations, we limited our analysis to a single biomedical subdomain.

### Impact of benchmark calibration

Our model was calibrated with Japanese authors serving as the benchmark for non-native speakers. Using a single country, Japan, as the non-native benchmark does potentially introduce a certain bias. However, the comparably large proportion of NS data used resulted in a classification model driven primarily by NS-derived features, as evidenced by the comparably much higher classification rate of NS abstracts. Further evidence for this includes the finding that GI scores correlate significantly with two external measures associated with writing quality, one case-by-case (CLI) and the other on the aggregate country-to-country level (IELTS). These results confirm the robustness our GI model, particularly in terms of rank-order validity.

To further test this assumption, we reran our GI model to observe what, if any, changes would result from using China as the nNS benchmark instead of Japan (S2 and S3 Tables). For 17 out of the 27 countries, t-test results suggested that mean GI scores were indeed significantly different in the China-based model. However, on exclusion of China and Japan, these changes were found to be well within the range of +/- 5, with a mean change of -0.2 (95% CI: -2.1, 1.7). As demonstrated previously, this is well within the threshold within which no significant differences could be observed between countries with respect to GI score (Table 3). Were that to be the relevant threshold for this comparison, then only two other countries show a change in GI score that could be considered remarkable: South Korea and Poland. Japan continues to cluster among the poorest performers, with a mean GI score not significantly different from the bottom rank (China excluded). This finding lends strong support to the overall rank-order validity of the GI scoring model demonstrated herein, while also underscoring the need for further research to determine the distribution characteristics most relevant for accurately interpreting differences in GI score, with respect to the key writing features characteristic of a given domain or subspeciality.

As a further control, our GI model was applied to a supplementary corpus that included an additional 16,952 abstracts in the field of anesthesiology. This experiment was inconclusive, with a classification precision of only 60.2% with respect to NS. We then combined the two corpora to explore what, if any, performance increase could be had by increasing the size and scope of sample. Overall results were found to be broadly similar to our original model (S4 Table): using the same parameters as our initial GI model, this combined corpus generated a GI model with recall that was twice that of our original model with respect to the nNS benchmark (53.9%). However, precision was found to be substantially lower (68.2%). These results suggest that regardless of the sample scope and size, the selection of benchmark has minimal overall impact with respect to the discriminatory power of this GI model.

### Impact of ethnic diversity within NS countries

Another consideration is that significant numbers of nNS researchers are affiliated with institutions based in NS countries. Our methodology assigned country of origin based on the affiliation of the first author. And while this choice was consistent with prior research and validated by the present, it is not unreasonable to suspect that the inadvertent inclusion of nNS speakers may have influenced the results somewhat. Indeed, our results found the aggregate scores of the NS countries to be substantially lower than the theoretical maximum (100). This would indicate a significant pool of sub-ideal submissions, even among the NS countries. Unfortunately, the present study included no mechanism to disambiguate country-of-origin and first author affiliation, or to explore the impact of (presumed) ethnicity on GI score. One possible route for further exploration would be a manual inspection of such low performing abstracts.

In this study, a GI score of 90 was found to be the cutoff for the 95th percentile. Thus abstracts assigned a score of 90 or more could be described to be “superior” in terms of fluency, as determined by the GI model. When examining the percentage of abstracts with such superior GI scores per country, Israel and the Netherlands each possessed a larger percentage of superior abstracts compared to the UK (Table 6). This result calls into question what, if any bias, may have been introduced due to the disproportionately large representation of US-based authors. However, given that the country with the largest proportion of superior abstracts was Australia and not the US, we find this hypothesis difficult to support. These findings were not tested for significance. Further research will be needed to better understand the attributes most closely associated with superior texts.

**Table 6:** Count/percentage of abstracts with GI score falling in 95%-ile.

Country	Count	Percentage
USA	203	10%
Canada	21	8%
UK	14	5%
The Netherlands	11	7%
Israel	10	7%
Australia	8	11%
Italy	5	2%
France	4	3%
India	4	3%
Sweden	2	2%
Denmark	2	4%
Germany	2	1%
Greece	1	1%
Turkey	1	0%
Switzerland	1	2%
Austria	1	2%
Norway	1	2%
**Total**	**291**	**5%**

Percentage of abstracts with “superior” scores within each country. “Superior” defined to be scores falling within the 95%-ile (i.e., greater than 89). Countries with no abstracts within the 95%-ile have not been included in this table.

### GI score interpretation

Despite the limitations discussed herein, our GI model was found to be quite robust. This study included no pre-selected positive control by which to evaluate our GI model. Some possible options might have been editorials from top journals, articles written by professional science NS writers, or selecting samples from a recognized authority in the field. However, it would have been difficult and methodologically unsound to arbitrarily declare any one writing sample to be deserving of a perfect GI score (100). For nNS, however, the implications are clear: there may indeed be no objective standard or abstractable ideal after which once should pattern their writing. English has long been considered a “living language” [69], one implication of which may be that everyone—NS and nNS alike—must continuously remain conscious of the trends driving discourse within their field. Our GI model provides nNS researchers with a clear indication of the language associated with such trends. Further research will be needed to better characterize the full range of language-related attributes with potential to affect publication results within a given field.

### Potential applications and benefits

The GI scoring system, as presented here, has many potential applications. For examples, nNS researchers might use such a system to evaluate the quality of professional editing services. As we have demonstrated, even with the benefit of such services, nNS researchers are still more likely to submit abstracts with substandard or field-inappropriate usage. By using a system based on such a GI scoring model, nNS researchers could compare GI scores before and after editing to objectively quantify editing value. Likewise, editors could use GI scoring to demonstrate the value of their work. A more advanced application might highlight the terms and phrases within a manuscript detrimental to the GI score. Another application might involve the identification and retrieval of higher-scoring abstracts with similar content, as determined by cluster analysis. This would provide researchers and editors alike with appropriate examples of superior language.

## Conclusion

Our resulting Genuine Index model was able to detect, with a high degree of reliability, subtle differences between the terms and phrasing used by native and non-native speakers in peer reviewed journals, in the field of pediatric oncology. This finding is especially remarkable given that the peer review and editing process is meant to homogenize research output with respect to English and field-specific language. And our findings do not repudiate this notion. Instead, our findings show that even when the language used is technically correct, there may still be some phrasing or usage conventions present that impact the readability and, ultimately, the dissemination reach of the scientific achievements of biomedical researchers, regardless of background.

## Supporting information

S1 Fig Summary of GI generation workflow. Data is fed to three sub-workflows: a) labeled data is used to generate the GI model and related outputs, b) generated GI model is applied to all data to assign scores, c) original input data is merged with generated data and formatted.
(TIFF)

S2 Fig GI score distribution by country. GI score distribution (light blue solid bars) confirmed to be approximately normal for each country and consistent with the overall distribution characteristics (gray shaded bars). The x axis of each histogram denotes the GI scores ranging 13–96, in discrete units (bins) of 5. The y axis shows the relative incidence of each bin within each respective distribution.
(TIFF)

S1 Table Comparison of GI scores between countries, with significance tests. (DOCX)

S2 Table GI score difference (China—Japan). (DOCX)

S3 Table Identification of homogenous subsets among countries w.r.t. GI score (Validation model using China as the nNS benchmark). (DOCX)

S4 Table Genuine Index model performance (validation corpus combining original pediatric oncology data set and validation-only anesthesiology data set). (DOCX)
