# Supplementary figures and images for: Evaluating the lexico-grammatical differences in the writing of native and non-native speakers of English in peer-reviewed medical journals in the field of pediatric oncology: Creation of the genuine index scoring system
Source: PLoS One. 2017 Feb 17;12(2):e0172338. doi: 10.1371/journal.pone.0172338 (PMC5315297; doi:10.1371/journal.pone.0172338)

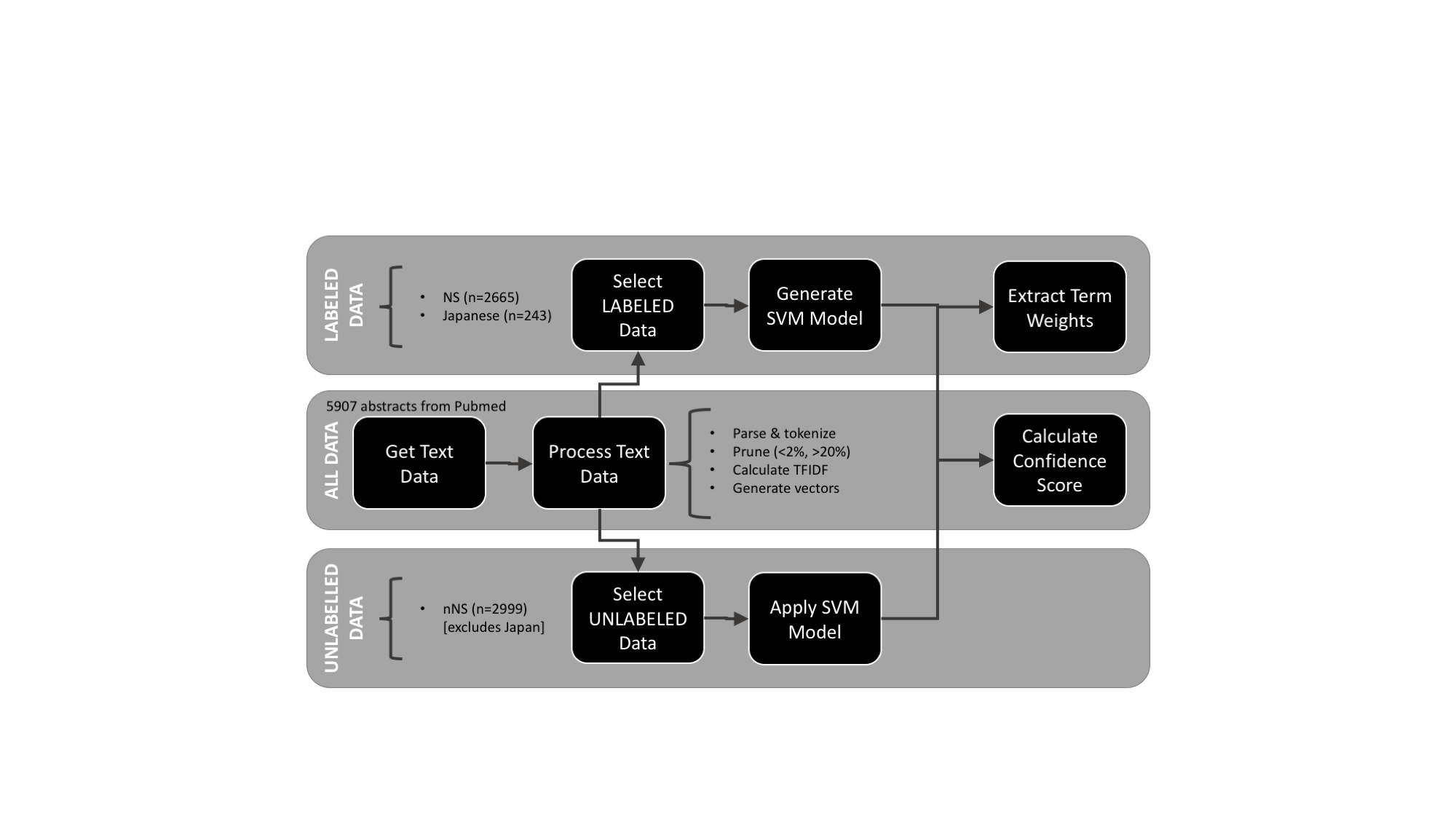

Supplement: S1 Fig — Data is fed to three sub-workflows: a) labeled data is used to generate the GI model and related outputs, b) generated GI model is applied to all data to assign scores, c) original input data is merged with generated data and formatted. (TIFF) [file pone.0172338.s001.tiff]

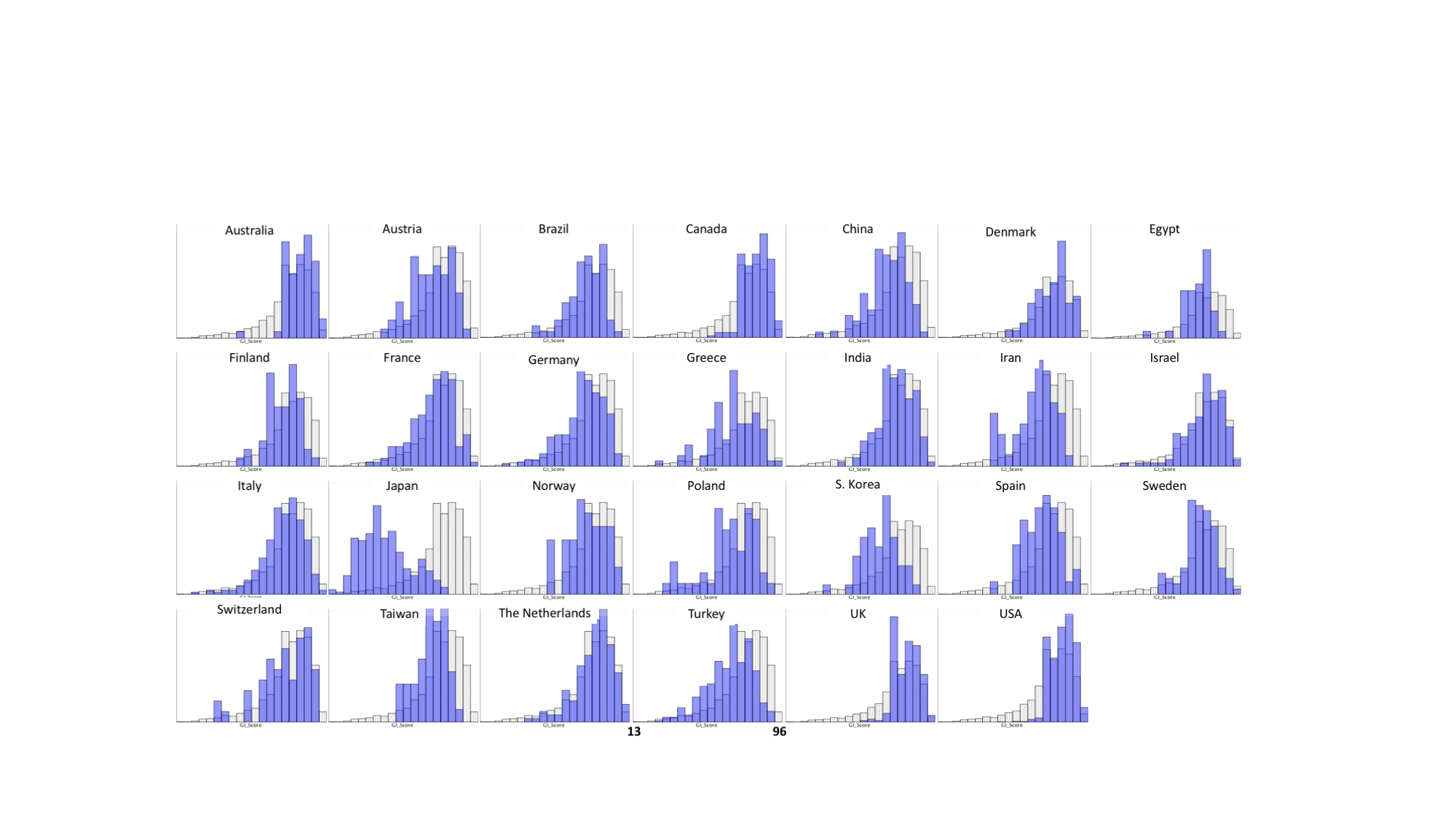

Supplement: S2 Fig — GI score distribution (light blue solid bars) confirmed to be approximately normal for each country and consistent with the overall distribution characteristics (gray shaded bars). The x axis of each histogram denotes the GI scores ranging 13–96, in discrete units (bins) of 5. The y axis shows the relative incidence of each bin within each respective distribution. (TIFF) [file pone.0172338.s002.tiff]
