# Supplementary material for: Evaluating the lexico-grammatical differences in the writing of native and non-native speakers of English in peer-reviewed medical journals in the field of pediatric oncology: Creation of the genuine index scoring system
Source: PLoS One. 2017 Feb 17;12(2):e0172338. doi: 10.1371/journal.pone.0172338 (PMC5315297; doi:10.1371/journal.pone.0172338)
Supplement: S1 Table — (DOCX) [file pone.0172338.s003.docx]

**S1 Table. Comparison of GI scores between countries, with significance tests.**

| Mean Difference (I-J) | Australia | Austria | Brazil | Canada | China | Denmark | Egypt | Finland | France | Germany | Greece | India | Iran | Israel | Italy | Japan | Norway | Poland | South Korea | Spain | Sweden | Switzerland | Taiwan | The  Netherlands | Turkey | UK | USA |
| --- | --- | --- | --- | --- | --- | --- | --- | --- | --- | --- | --- | --- | --- | --- | --- | --- | --- | --- | --- | --- | --- | --- | --- | --- | --- | --- | --- |
| Australia | - | 11.2* | 9.5* | -0.2 | 11.9* | 5 | 8.9* | 8.8* | 10.1* | 10.8* | 12.8* | 6.9* | 14.8* | 5.6* | 9.2* | 37* | 8.9* | 14.7* | 15.8* | 10.7* | 7.5* | 8.5* | 11.5* | 4.6 | 14.3* | 1.1 | -0.7 |
| Austria | -11.2* | - | -1.8 | -11.4* | 0.6 | -6.2 | -2.3 | -2.5 | -1.2 | -0.4 | 1.6 | -4.3 | 3.6 | -5.6* | -2 | 25.8* | -2.3 | 3.5 | 4.6 | -0.6 | -3.7 | -2.8 | 0.3 | -6.6* | 3 | -10.1* | -11.9* |
| Brazil | -9.5* | 1.8 | - | -9.6* | 2.4 | -4.5 | -0.5 | -0.7 | 0.6 | 1.3 | 3.3 | -2.5 | 5.3 | -3.9 | -0.2 | 27.5* | -0.6 | 5.3 | 6.3* | 1.2 | -1.9 | -1 | 2 | -4.9* | 4.8* | -8.4* | -10.2* |
| Canada | 0.2 | 11.4* | 9.6* | - | 12.1* | 5.2* | 9.1* | 8.9* | 10.2* | 11* | 13* | 7.1* | 15* | 5.8* | 9.4* | 37.2* | 9.1* | 14.9* | 16* | 10.8* | 7.7* | 8.7* | 11.7* | 4.8* | 14.5* | 1.3 | -0.5 |
| China | -11.9* | -0.6 | -2.4 | -12.1* | - | -6.9* | -2.9 | -3.1 | -1.8 | -1.1 | 0.9 | -4.9* | 2.9 | -6.3* | -2.6 | 25.1* | -3 | 2.9 | 3.9 | -1.2 | -4.3 | -3.4 | -0.4 | -7.3* | 2.4 | -10.8* | -12.6* |
| Denmark | -5 | 6.2 | 4.5 | -5.2* | 6.9* | - | 3.9 | 3.8 | 5.1 | 5.8* | 7.8* | 1.9 | 9.8* | 0.6 | 4.2 | 32* | 3.9 | 9.7* | 10.8* | 5.7 | 2.5 | 3.5 | 6.5 | -0.4 | 9.3* | -3.9 | -5.7* |
| Egypt | -8.9* | 2.3 | 0.5 | -9.1* | 2.9 | -3.9 | - | -0.2 | 1.1 | 1.9 | 3.9 | -2 | 5.8 | -3.3 | 0.3 | 28.1* | 0 | 5.8 | 6.9 | 1.7 | -1.4 | -0.5 | 2.6 | -4.3 | 5.3 | -7.8* | -9.6* |
| Finland | -8.8* | 2.5 | 0.7 | -8.9* | 3.1 | -3.8 | 0.2 | - | 1.3 | 2 | 4 | -1.8 | 6 | -3.2 | 0.5 | 28.2* | 0.1 | 6 | 7* | 1.9 | -1.2 | -0.3 | 2.7 | -4.2 | 5.5* | -7.7* | -9.5* |
| France | -10.1* | 1.2 | -0.6 | -10.2* | 1.8 | -5.1 | -1.1 | -1.3 | - | 0.8 | 2.8 | -3.1 | 4.7 | -4.4* | -0.8 | 26.9* | -1.1 | 4.7 | 5.7* | 0.6 | -2.5 | -1.6 | 1.4 | -5.4* | 4.2* | -8.9* | -10.8* |
| Germany | -10.8* | 0.4 | -1.3 | -11* | 1.1 | -5.8* | -1.9 | -2 | -0.8 | - | 2 | -3.9* | 4 | -5.2* | -1.6 | 26.2* | -1.9 | 3.9 | 5 | -0.2 | -3.3 | -2.3 | 0.7 | -6.2* | 3.5* | -9.7* | -11.5* |
| Greece | -12.8* | -1.6 | -3.3 | -13* | -0.9 | -7.8* | -3.9 | -4 | -2.8 | -2 | - | -5.9* | 2 | -7.2* | -3.6 | 24.2* | -3.9 | 1.9 | 3 | -2.1 | -5.3* | -4.3 | -1.3 | -8.2* | 1.5 | -11.7* | -13.5* |
| India | -6.9* | 4.3 | 2.5 | -7.1* | 4.9* | -1.9 | 2 | 1.8 | 3.1 | 3.9* | 5.9* | - | 7.9* | -1.3 | 2.3 | 30.1* | 2 | 7.8* | 8.9* | 3.7 | 0.6 | 1.5 | 4.6 | -2.3 | 7.3* | -5.8* | -7.6* |
| Iran | -14.8* | -3.6 | -5.3 | -15* | -2.9 | -9.8* | -5.8 | -6 | -4.7 | -4 | -2 | -7.9* | - | -9.2* | -5.5* | 22.2* | -5.9 | 0 | 1 | -4.1 | -7.3* | -6.3 | -3.3 | -10.2* | -0.5 | -13.7* | -15.5* |
| Israel | -5.6* | 5.6* | 3.9 | -5.8* | 6.3* | -0.6 | 3.3 | 3.2 | 4.4* | 5.2* | 7.2* | 1.3 | 9.2* | - | 3.6* | 31.4* | 3.3 | 9.1* | 10.2* | 5 | 1.9 | 2.9 | 5.9* | -1 | 8.7* | -4.5* | -6.3* |
| Italy | -9.2* | 2 | 0.2 | -9.4* | 2.6 | -4.2 | -0.3 | -0.5 | 0.8 | 1.6 | 3.6 | -2.3 | 5.5* | -3.6* | - | 27.8* | -0.3 | 5.5* | 6.6* | 1.4 | -1.7 | -0.8 | 2.3 | -4.6* | 5* | -8.1* | -9.9* |
| Japan | -37* | -25.8* | -27.5* | -37.2* | -25.1* | -32* | -28.1* | -28.2* | -26.9* | -26.2* | -24.2* | -30.1* | -22.2* | -31.4* | -27.8* | - | -28.1* | -22.3* | -21.2* | -26.3* | -29.5* | -28.5* | -25.5* | -32.4* | -22.7* | -35.9* | -37.7* |
| Norway | -8.9* | 2.3 | 0.6 | -9.1* | 3 | -3.9 | 0 | -0.1 | 1.1 | 1.9 | 3.9 | -2 | 5.9 | -3.3 | 0.3 | 28.1* | - | 5.8 | 6.9 | 1.7 | -1.4 | -0.4 | 2.6 | -4.3 | 5.4 | -7.8* | -9.6* |
| Poland | -14.7* | -3.5 | -5.3 | -14.9* | -2.9 | -9.7* | -5.8 | -6 | -4.7 | -3.9 | -1.9 | -7.8* | 0 | -9.1* | -5.5* | 22.3* | -5.8 | - | 1.1 | -4.1 | -7.2* | -6.3 | -3.2 | -10.1* | -0.5 | -13.6* | -15.4* |
| South Korea | -15.8* | -4.6 | -6.3* | -16* | -3.9 | -10.8* | -6.9 | -7* | -5.7* | -5 | -3 | -8.9* | -1 | -10.2* | -6.6* | 21.2* | -6.9 | -1.1 | - | -5.1 | -8.3* | -7.3* | -4.3 | -11.2* | -1.5 | -14.7* | -16.5* |
| Spain | -10.7* | 0.6 | -1.2 | -10.8* | 1.2 | -5.7 | -1.7 | -1.9 | -0.6 | 0.2 | 2.1 | -3.7 | 4.1 | -5 | -1.4 | 26.3* | -1.7 | 4.1 | 5.1 | - | -3.1 | -2.2 | 0.8 | -6* | 3.6 | -9.6* | -11.4* |
| Sweden | -7.5* | 3.7 | 1.9 | -7.7* | 4.3 | -2.5 | 1.4 | 1.2 | 2.5 | 3.3 | 5.3* | -0.6 | 7.3* | -1.9 | 1.7 | 29.5* | 1.4 | 7.2* | 8.3* | 3.1 | - | 0.9 | 4 | -2.9 | 6.7* | -6.4* | -8.2* |
| Switzerland | -8.5* | 2.8 | 1 | -8.7* | 3.4 | -3.5 | 0.5 | 0.3 | 1.6 | 2.3 | 4.3 | -1.5 | 6.3 | -2.9 | 0.8 | 28.5* | 0.4 | 6.3 | 7.3* | 2.2 | -0.9 | - | 3 | -3.9 | 5.8* | -7.4* | -9.2* |
| Taiwan | -11.5* | -0.3 | -2 | -11.7* | 0.4 | -6.5 | -2.6 | -2.7 | -1.4 | -0.7 | 1.3 | -4.6 | 3.3 | -5.9* | -2.3 | 25.5* | -2.6 | 3.2 | 4.3 | -0.8 | -4 | -3 | - | -6.9* | 2.8 | -10.4* | -12.2* |
| The Netherlands | -4.6 | 6.6* | 4.9* | -4.8* | 7.3* | 0.4 | 4.3 | 4.2 | 5.4* | 6.2* | 8.2* | 2.3 | 10.2* | 1 | 4.6* | 32.4* | 4.3 | 10.1* | 11.2* | 6* | 2.9 | 3.9 | 6.9* | - | 9.7* | -3.5* | -5.3* |
| Turkey | -14.3* | -3 | -4.8* | -14.5* | -2.4 | -9.3* | -5.3 | -5.5* | -4.2* | -3.5* | -1.5 | -7.3* | 0.5 | -8.7* | -5* | 22.7* | -5.4 | 0.5 | 1.5 | -3.6 | -6.7* | -5.8* | -2.8 | -9.7* | - | -13.2* | -15* |
| UK | -1.1 | 10.1* | 8.4* | -1.3 | 10.8* | 3.9 | 7.8* | 7.7* | 8.9* | 9.7* | 11.7* | 5.8* | 13.7* | 4.5* | 8.1* | 35.9* | 7.8* | 13.6* | 14.7* | 9.6* | 6.4* | 7.4* | 10.4* | 3.5* | 13.2* | - | -1.8 |
| USA | 0.7 | 11.9* | 10.2* | 0.5 | 12.6* | 5.7* | 9.6* | 9.5* | 10.8* | 11.5* | 13.5* | 7.6* | 15.5* | 6.3* | 9.9* | 37.7* | 9.6* | 15.4* | 16.5* | 11.4* | 8.2* | 9.2* | 12.2* | 5.3* | 15* | 1.8 | - |

ANOVA post-hoc analysis of differences between means. Difference between row and column shown. Significance tests conducted using Tukey HSD post-hoc method. Statistical differences highlighted.
