# Supplementary material for: Evaluating the lexico-grammatical differences in the writing of native and non-native speakers of English in peer-reviewed medical journals in the field of pediatric oncology: Creation of the genuine index scoring system
Source: PLoS One. 2017 Feb 17;12(2):e0172338. doi: 10.1371/journal.pone.0172338 (PMC5315297; doi:10.1371/journal.pone.0172338)
Supplement: S2 Table — (DOCX) [file pone.0172338.s004.docx]

S2 Table. GI score difference (China – Japan)

| **Paired Samples Test** | | | | |
| --- | --- | --- | --- | --- |
|  | Paired Differences | | | Sig. (2-tailed) |
| GI score differences | Mean | 95% Confidence Interval of the Difference | |  |
| China - Japan |  | Lower | Upper |  |
| Australia | -4.70 | -6.40 | -3.00 | 0.00 |
| Austria | 3.35 | 0.89 | 5.81 | 0.01 |
| Brazil | 0.47 | -1.57 | 2.52 | 0.65 |
| Canada | -4.54 | -5.30 | -3.79 | 0.00 |
| China | -13.40 | -15.43 | -11.37 | 0.00 |
| Denmark | -2.31 | -4.73 | 0.11 | 0.06 |
| Egypt | -2.57 | -5.47 | 0.33 | 0.08 |
| Finland | 0.54 | -1.84 | 2.92 | 0.65 |
| France | 1.77 | 0.19 | 3.34 | 0.03 |
| Germany | 2.77 | 1.44 | 4.09 | 0.00 |
| Greece | 3.66 | 0.91 | 6.41 | 0.01 |
| India | -2.23 | -3.87 | -0.59 | 0.01 |
| Iran | 1.72 | -1.36 | 4.80 | 0.27 |
| Israel | -2.86 | -4.55 | -1.17 | 0.00 |
| Italy | 1.82 | 0.47 | 3.18 | 0.01 |
| Japan | 26.88 | 25.25 | 28.50 | 0.00 |
| Norway | -0.21 | -3.32 | 2.89 | 0.89 |
| Poland | 5.28 | 2.33 | 8.24 | 0.00 |
| South Korea | 5.23 | 2.36 | 8.09 | 0.00 |
| Spain | 1.15 | -1.57 | 3.88 | 0.40 |
| Sweden | -0.70 | -2.31 | 0.91 | 0.39 |
| Switzerland | 1.48 | -1.88 | 4.84 | 0.38 |
| Taiwan | -0.04 | -2.99 | 2.90 | 0.98 |
| The Netherlands | -3.14 | -4.61 | -1.67 | 0.00 |
| Turkey | 3.52 | 2.45 | 4.60 | 0.00 |
| UK | -3.70 | -4.52 | -2.88 | 0.00 |
| USA | -4.53 | -4.78 | -4.28 | 0.00 |
