# Supplementary material for: Evaluating the lexico-grammatical differences in the writing of native and non-native speakers of English in peer-reviewed medical journals in the field of pediatric oncology: Creation of the genuine index scoring system
Source: PLoS One. 2017 Feb 17;12(2):e0172338. doi: 10.1371/journal.pone.0172338 (PMC5315297; doi:10.1371/journal.pone.0172338)
Supplement: S3 Table — (DOCX) [file pone.0172338.s005.docx]

S3 Table. Identification of homogenous subsets among countries w.r.t. GI score (Validation model using China as the nNS benchmark)

|  | Homogenous Subsets Tukey HSD_a,b_ | | | | | | | | | | | |
| --- | --- | --- | --- | --- | --- | --- | --- | --- | --- | --- | --- | --- |
| Country |  | 1 | 2 | 3 | 4 | 5 | 6 | 7 | 8 | 9 | 10 |  |
| China | 103 | 55.6 |  |  |  |  |  |  |  |  |  |  |
| Iran | 54 |  | 68.4 |  |  |  |  |  |  |  |  |  |
| Taiwan | 45 |  | 68.7 | 68.7 |  |  |  |  |  |  |  |  |
| Turkey | 476 |  | 69.7 | 69.7 | 69.7 |  |  |  |  |  |  |  |
| Egypt | 42 |  | 69.9 | 69.9 | 69.9 | 69.9 |  |  |  |  |  |  |
| South Korea | 48 |  | 70.3 | 70.3 | 70.3 | 70.3 | 70.3 |  |  |  |  |  |
| Japan | 244 |  | 70.8 | 70.8 | 70.8 | 70.8 | 70.8 | 70.8 |  |  |  |  |
| Poland | 53 |  | 70.8 | 70.8 | 70.8 | 70.8 | 70.8 | 70.8 |  |  |  |  |
| Greece | 86 |  | 70.8 | 70.8 | 70.8 | 70.8 | 70.8 | 70.8 |  |  |  |  |
| Spain | 46 |  | 71.1 | 71.1 | 71.1 | 71.1 | 71.1 | 71.1 |  |  |  |  |
| India | 136 |  |  | 71.2 | 71.2 | 71.2 | 71.2 | 71.2 |  |  |  |  |
| Brazil | 78 |  |  | 71.4 | 71.4 | 71.4 | 71.4 | 71.4 |  |  |  |  |
| Norway | 42 |  |  |  | 71.9 | 71.9 | 71.9 | 71.9 | 71.9 |  |  |  |
| Finland | 54 |  |  |  | 72.0 | 72.0 | 72.0 | 72.0 | 72.0 |  |  |  |
| Israel | 139 |  |  |  | 72.4 | 72.4 | 72.4 | 72.4 | 72.4 |  |  |  |
| Austria | 63 |  |  |  |  | 72.6 | 72.6 | 72.6 | 72.6 |  |  |  |
| Sweden | 87 |  |  |  |  |  | 72.7 | 72.7 | 72.7 |  |  |  |
| France | 145 |  |  |  |  |  | 72.7 | 72.7 | 72.7 |  |  |  |
| Denmark | 55 |  |  |  |  |  | 72.9 | 72.9 | 72.9 |  |  |  |
| The Netherlands | 161 |  |  |  |  |  | 72.9 | 72.9 | 72.9 |  |  |  |
| Germany | 273 |  |  |  |  |  | 73.1 | 73.1 | 73.1 |  |  |  |
| Italy | 283 |  |  |  |  |  |  | 73.4 | 73.4 | 73.4 |  |  |
| Switzerland | 54 |  |  |  |  |  |  |  | 74.4 | 74.4 | 74.4 |  |
| UK | 262 |  |  |  |  |  |  |  |  | 75.9 | 75.9 |  |
| Australia | 71 |  |  |  |  |  |  |  |  |  | 76.3 |  |
| Canada | 272 |  |  |  |  |  |  |  |  |  | 76.4 |  |
| USA | 2085 |  |  |  |  |  |  |  |  |  | 76.9 |  |
|  | Sig. |  | 1.00 | 0.08 | 0.08 | 0.07 | 0.06 | 0.06 | 0.10 | 0.12 | 0.11 | 0.17 |
