# Supplementary material for: Evaluating the lexico-grammatical differences in the writing of native and non-native speakers of English in peer-reviewed medical journals in the field of pediatric oncology: Creation of the genuine index scoring system
Source: PLoS One. 2017 Feb 17;12(2):e0172338. doi: 10.1371/journal.pone.0172338 (PMC5315297; doi:10.1371/journal.pone.0172338)
Supplement: S4 Table — (DOCX) [file pone.0172338.s006.docx]

**S4 Table. Genuine Index model performance (validation corpus combining original pediatric oncology data set and validation-only anesthesiology data set)**

|  | True JPN  (n=1448) | True NS  (n=10544) | Precision |
| --- | --- | --- | --- |
| Predicted JPN (n=1143) | 780 | 363 | 68.2% |
| Predicted NS (n=10849) | 668 | 10181 | 93.8% |
| Recall | 53.9% | 96.6% |  |
